# *Lithospermum erythrorhizon* and *Forsythia suspensa* Prevent Collagen Degradation and Maintain Skin Hydration by Regulating MMPs and HAS2/HYAL1 Signaling

**DOI:** 10.3390/molecules30051083

**Published:** 2025-02-27

**Authors:** Xiangji Jin, Qiwen Zheng, Trang Thi Minh Nguyen, Se-Jig Park, Gyeong-Seon Yi, Su-Jin Yang, Tae-Hoo Yi

**Affiliations:** 1 Department of Dermatology, School of Medicine, Graduate School, Kyung Hee University, 26 Kyungheedae-ro, Dong-daemun, Seoul 02447, Republic of Korea; hyanghe112@khu.ac.kr; 2 Graduate School of Biotechnology, Kyung Hee University, 1732 Deogyeong-daero, Giheung-gu, Yongin-si 17104, Republic of Korea; zhengqiwen@khu.ac.kr (Q.Z.); trangnguyen@khu.ac.kr (T.T.M.N.); tpwlt@khu.ac.kr (S.-J.P.); stella@khu.ac.kr (S.-J.Y.); 3 Department of Convergent Biotechnology and Advanced Materials Engineering, Graduate School, Kyung Hee University, Yongin 17104, Republic of Korea; ks010924@khu.ac.kr

**Keywords:** *Lithospermum erythrorhizon*, *Forsythia suspensa*, hyaluronic acid, matrix metalloproteinases (MMPs)

## Abstract

The growing demand for aesthetic enhancement has driven the development of anti-aging cosmetics, with natural compound-based formulations emerging as a new trend to enhance efficacy. This study aims to develop a 30% ethanol extract of a 1:1 mixture of *Lithospermum erythrorhizon* and *Forsythia suspensa* (LF) as a potential material for combating UVB-induced skin aging. The bioactive components of LF extract were identified via HPLC. Antioxidant efficacy (DPPH, ABTS, and SOD) and the inhibitory effects on ROS production in cells were evaluated using flow cytometry. MMPs’ expressions were analyzed via RT-PCR, while TGF-β/Smad, ERK/AP-1, and HAS2/HYAL1 pathways were examined via ELISA and Western blot. Research findings indicate that LF effectively scavenges reactive oxygen species and enhances the activation of TGF-β signaling, promoting the synthesis of PIP (Procollagen Type I C-Peptide). Collagen degradation was mitigated through the inhibition of the AP-1 pathway, which regulates the expression of MMPs, and by suppressing the expression of TIMP. Additionally, modulation of the HAS2/HYAL1 signaling axis ensures a balanced regulation of hyaluronic acid (HA) synthesis and degradation, thereby contributing to the maintenance of collagen integrity and skin hydration. In conclusion, LF has exhibited significant protective effects against demonstrated anti-aging properties, highlighting its potential as a novel therapeutic agent in cosmetic formulations targeting aging.

## 1. Introduction

Ultraviolet B (UVB) radiation is a major contributor to skin photoaging, initiating a cascade of cellular responses [1]. One of the first events following UVB exposure is the generation of reactive oxygen species (ROSs), which triggers oxidative stress in the skin [2]. This oxidative stress activates tissue inhibitors of metalloproteinases (TIMPs) that play a crucial role in regulating the activity of matrix metalloproteinases (MMPs). TIMPs typically inhibit MMPs, which are involved in the breakdown of extracellular matrix (ECM) components like collagen and elastin [3]. However, in photoaging, the balance between TIMPs and MMPs is disrupted. ROS-induced upregulation of MMPs and the downregulation or inactivation of TIMPs lead to uncontrolled MMP activity, resulting in the degradation of collagen fibers and the deterioration of the skin structure [4]. This imbalance contributes to the visible signs of photoaging, such as wrinkles, loss of elasticity, and sagging skin [5]. Consequently, maintaining proper TIMP expression is essential for skin integrity and the prevention of UV-induced ECM breakdown.

Following ROS formation, the ERK/MAPK pathway is activated in response to UVB radiation. Extracellular signal-regulated kinases (ERK1/2), part of the MAPK family, plays a crucial role in cellular responses to UV-induced damage and other stressors by regulating processes such as cell survival and differentiation [6]. In photoaging, the activation of ERK1/2 leads to the phosphorylation and activation of activator protein 1 (AP-1), a transcription factor complex that includes c-Fos and c-Jun. Phosphorylation of c-Fos (P-c-Fos) enhances its transcriptional activity, which, in turn, stimulates the expression of MMPs, such as MMP1, MMP-3, and MMP-9 [7]. These MMPs degrade ECM components, including collagen and elastin fibers, contributing to the loss of skin firmness and elasticity. The ERK/AP-1 signaling cascade plays a critical role in the progression of photoaging, as it facilitates ECM degradation through the induction of MMPs. This pathway establishes a feedback loop where MMP-mediated ECM breakdown perpetuates the skin’s aging process following UVB exposure [8,9].

TGF-β (Transforming Growth Factor-beta) signaling is crucial for maintaining ECM homeostasis, particularly in regulating collagen synthesis [10]. TGF-β activates the SMAD2/3 pathway, which translocates to the nucleus to regulate gene expression associated with ECM components, including collagen [11]. In photoaging, UV exposure induces oxidative stress that results in the upregulation of SMAD7, an inhibitor of SMAD2/3 signaling. Increased SMAD7 levels impair the activation of SMAD2/3, reducing collagen synthesis and compromising ECM integrity. This disruption contributes to the hallmark features of photoaging, such as wrinkles, sagging, and loss of skin elasticity [12]. The dysregulation of TGF-beta signaling and its downstream effectors, particularly the increased expression of SMAD7, plays a central role in ECM degradation and the acceleration of photoaging processes [13].

HA is crucial for skin hydration and elasticity, maintaining moisture balance in the ECM. UV exposure disrupts HA synthesis and accelerates its degradation, leading to signs of photoaging like dryness, sagging, and reduced elasticity [14]. The synthesis of HA is regulated by hyaluronic acid synthases (HASs), while its breakdown is mediated by hyaluronidases (HYALs). UV radiation upregulates hyaluronidase activity and inhibits HA production, reducing the skin’s moisture retention and weakening ECM structure, contributing to wrinkles and fine lines. Additionally, UV-induced oxidative stress increases ROSs, damaging HA and related enzymes. Maintaining a balance between HA synthesis and degradation is essential to prevent UV-induced skin aging [15,16,17].

*Lithospermum erythrorhizon* and *Forsythia suspensa*, have demonstrated promising effects in mitigating photoaging [18,19]. *Lithospermum erythrorhizon* contains shikonin, a compound with antioxidant, anti-inflammatory, and collagen-stimulating properties [20]. Research has shown that shikonin reduces oxidative stress induced by UVB radiation and protects against collagen degradation, thereby enhancing skin elasticity and reducing wrinkles. For instance, a study highlighted the ability of shikonin to improve skin elasticity and prevent UVB-induced skin aging [21]. Similarly, *Forsythia suspensa* has been found to exhibit anti-inflammatory and skin-healing effects. Forsythoside A, a bioactive compound in *Forsythia suspensa*, has shown promise in promoting collagen synthesis and improving the skin barrier function, making it an effective agent for skin rejuvenation and anti-aging [22]. By modulating the expression of cytokines and promoting collagen production, these herbal compounds offer potential therapeutic strategies to counteract UV-induced skin aging and enhance skin regeneration [23].

Despite extensive research on the individual effects of these natural compounds, no studies have yet explored the synergistic effects of combining *Lithospermum erythrorhizon* and *Forsythia suspensa* in the context of UVB-induced skin aging. Given the complementary mechanisms of these two compounds, it is imperative to investigate their combined potential for preventing and treating UVB-induced photoaging. Thus, we propose to conduct a study that examines the efficacy of these two natural products in mitigating the adverse effects of UVB radiation on skin, with the goal of providing a novel, integrative approach for skin protection and rejuvenation.

## 2. Results

### 2.1. Analysis of the LF Ethanolic Extract Compounds

**Figure 1:**
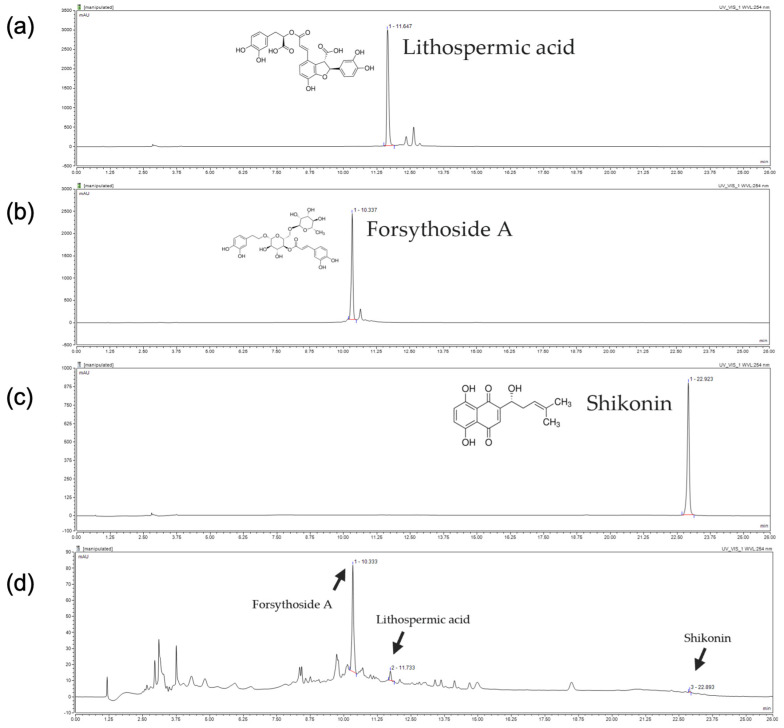
HPLC analysis of (**a**) Lithospermic acid, (**b**) Forsythoside A, and (**c**) Shikonin standards. (**d**) The content of LF extract.

To prepare LF samples, we extracted dried Lithospermum erythrorhizon (50 g) and Forsythia suspensa powder (50 g) using 30% ethanol, resulting in 29.86 g of LF crude product. As illustrated in Figure 1, lithospermic acid, forsythoside A, and shikonin were identified at retention times of 10.333, 11.733, and 22.893 min, respectively, by correlating these values with those of the reference standards.

### 2.2. Quantification of the Total Polyphenol and Flavonoid Content in LF: Data Are Expressed as the Mean ± SD

Phenolic compounds and flavonoids are bioactive phytochemicals that play a pivotal role in antioxidant mechanisms by mediating redox reactions. These bioactive compounds are essential in mitigating oxidative stress by neutralizing free radicals and regulating diverse biochemical signaling pathways [24]. The Total Phenolic Content (TPC) of LF extract was measured as 350.48 ± 4.59 mg GAE/g extract when using deuterium-depleted water and 1340 ± 2.19 mg GAE/g extract when using dimethyl sulfoxide. Similarly, the Total Flavonoid Content (TFC) of LF extract was found to be 313.2 ± 7.09 mg QE/g extract with deuterium-depleted water and 144.2 ± 2.03 mg QE/g extract with dimethyl sulfoxide.

### 2.3. Evaluation of Antioxidant Activity of LF

**Figure 2:**
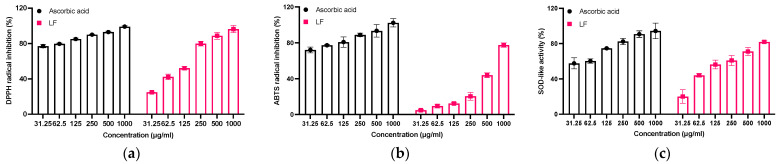
(**a**) DPPH (**b**) ABTS, and (**c**) SOD-like activity of LF. The results are shown as the mean ± SD of three independent experiments.

To investigate the antioxidative potential of LF, experiments were conducted using DPPH, ABTS, and SOD-like activity. The data demonstrated that LF enhanced radical scavenging activity in a concentration-dependent fashion, as observed in both DPPH and ABTS assays, with IC_50_ values of 89.48 μg/mL and 118.4 μg/mL. Additionally, the SOD-like activity was observed at 134.0 μg/mL. However, in the ABTS assay, when compared with the positive control AA, the ABTS+ radical scavenging activity was not significant, and its antioxidant capacity was found to be relatively poor. This suggests that LF does not play a major role in scavenging ABTS+ radicals. However, in the other two assays, DPPH and SOD-like, LF demonstrated notable antioxidant activity (Figure 2).

### 2.4. Toxicity and Cytoprotective Effects of LF on UVB-Irradiated HaCaT Cells

**Figure 3:**
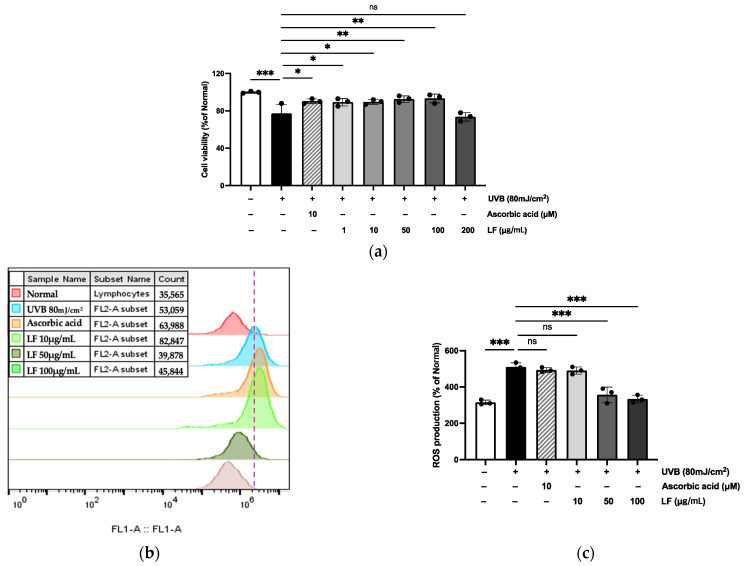
Analysis of cytotoxicity and intracellular ROS generation in LF. (**a**) Assessment of cell viability. (**b**) Intracellular ROS levels are plotted against DCFH-DA levels detected by the FL-1 channel. (**c**) Representation of relative intensity histogram. Data are shown as means ± SD of three independent experiments. Statistical significance was assessed using one-way ANOVA. * *p* < 0.05, ** *p* < 0.01, *** *p* < 0.001 compared with the only UVB-exposed group; ns indicates no significant difference.

The toxicity of LF on HaCaT cells was evaluated using the MTT assay. The results, shown in Figure 3, revealed that at 200 μg/mL, there was cytotoxicity compared to the UVB-induced control, and therefore, the highest concentration used in all experiments was determined to be 100 μg/mL. Additionally, intracellular ROS levels were quantified using flow cytometry, and the ROS scavenging potential of LF was assessed through the DCFH-DA fluorescence assay. The results demonstrated that LF treatment significantly attenuated the UVB-induced elevation of fluorescence intensity, reducing it to 51.9%.

### 2.5. Regulation of TIMP-1, HYAL1, and PIP Expression in HaCaT Cells

**Figure 4:**
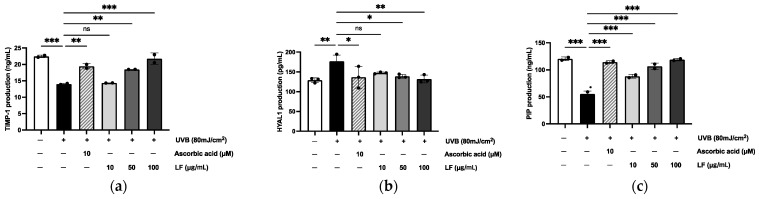
Inhibitory effect of LF on the production of TIMP-1 (**a**), HYAL1 (**b**), and PIP (**c**) in UVB-irradiated HaCaT cells. Data represent mean ± SD from triplicate wells. Statistical significance was assessed using one-way ANOVA. * *p* < 0.05, ** *p* < 0.01, *** *p* < 0.001 compared with the only UVB-exposed group; ns indicates no significant difference.

To observe the inhibitory effect of LF, the protein levels of TIMP-1, HYAL1, and PIP (Procollagen Type I C-Peptide) secreted into the culture medium by UVB-exposed cells were quantified using an enzyme-linked immunosorbent assay. In comparison to the levels secreted by the untreated group, UVB irradiation induced a 54.96% increase in TIMP-1 levels, a 50.17% increase in PIP protein levels, and a 25.49% increase in HYAL1 secretion in the control group exposed to UVB (Figure 4).

### 2.6. MMP-1, MMP-2, MMP-3, MMP-9 Regulation Effect on UVB-Irradiated HaCaT Cells

**Figure 5:**
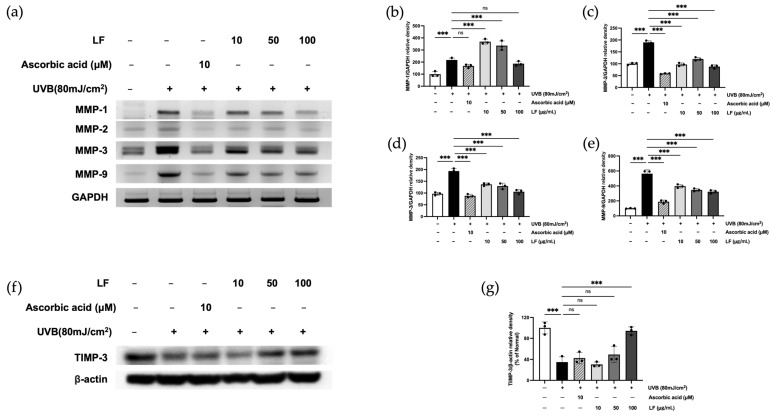
Expression of MMP mRNA, TIMP-3 proteins in response to LF was analyzed. (**a**) Evaluation of MMP mRNA levels; quantification of the band intensities (**b**–**e**). Evaluation of TIMP-3 protein levels (**f**); quantification of the band intensities (**g**). Data represent mean ± SD from triplicate wells. Statistical significance was assessed using one-way ANOVA. *** *p* < 0.001 compared with the only UVB-exposed group; ns indicates no significant difference.

Elevated MMP expression and reduced levels of procollagen and TGF-β are characteristic features of photoaging [18]. To evaluate the impact of LF on MMPs in UVB-irradiated HaCaT cells, mRNA expression was assessed via RT-PCR. In particular, when compared to UVB-exposed cells, treatment with 100 μg/mL LF reduced MMPs mRNA expression by 13.94%, 53.68%, 45.41%, and 42.78%, respectively (Figure 5a–e). As shown in Figure 5f,g, the expression of TIMP-3 has been proven to increase in a dose-dependent manner following LF treatment in response to various age-related stressors, including oxidative damage and UVB exposure. This increase in TIMP-3 expression likely acts as a protective mechanism to regulate the activity of MMPs, which are responsible for degrading ECM components such as collagen during skin aging.

### 2.7. ERK Signaling and AP-1 Activation by LF Treatment

**Figure 6:**
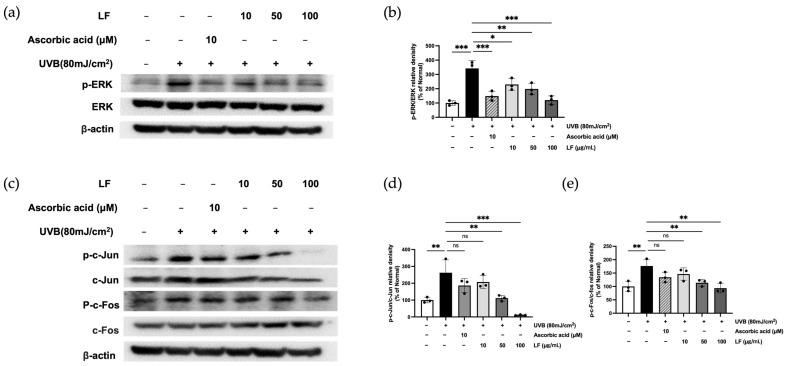
LF inhibited the phosphorylation of ERK/AP-1-signaling protein levels in UVB-exposed HaCaT cells: (**a**) Phosphorylated and non-phosphorylated members of the ERK. (**c**) AP-1 complex (c-fos and c-jun) protein levels; the band intensities (**b**,**d**,**e**). Data represent mean ± SD from triplicate wells. Statistical significance was assessed using one-way ANOVA. * *p* < 0.05, ** *p* < 0.01, *** *p* < 0.001 compared with the only UVB-exposed group; ns indicates no significant difference.

To investigate the influence of LF on the ERK/AP-1 signaling axis in UVB-exposed keratinocytes, we measured the expression levels of phosphorylated ERK (p-ERK), JNK (p-JNK), p38 (p-p38), c-fos (p-c-fos), and c-jun (p-c-jun) using Western blot analysis (Figure 6). UVB irradiation significantly upregulated the phosphorylation of these signaling molecules in HaCaT cells, indicating activation of the ERK/AP-1 pathway. However, treatment with LF led to a dose-dependent suppression of the phosphorylation of these key proteins, suggesting that LF inhibits the activation of the ERK/AP-1 signaling cascade in response to UVB-induced stress. These results suggest that LF may protect against UVB-induced skin damage by modulating the ERK/AP-1 signaling pathway.

### 2.8. LF Treatment on TGF-β/Smad7 Signaling and HYAL and HAS Regulation Pathways

**Figure 7:**
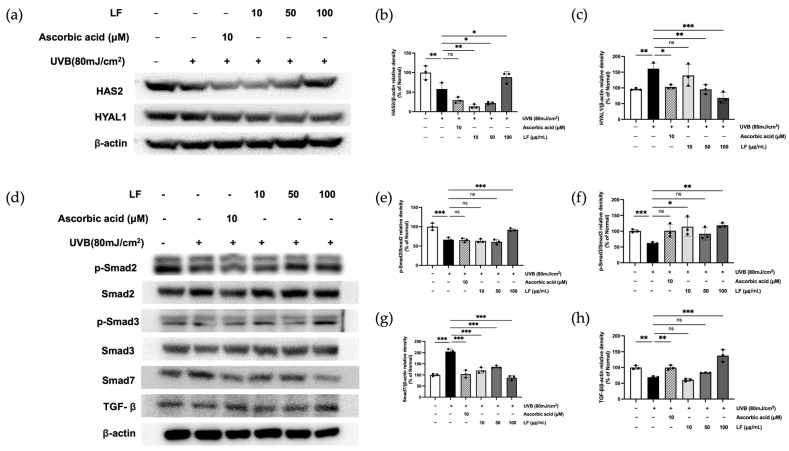
TGF-β/Smad signaling pathway, HYAL, and HAS proteins in response to LF was analyzed in UVB-exposed HaCaT cells: (**a**) Evaluation of HYAL1 and HAS protein levels. (**d**) Evaluation of p-Smad2/Smad2, p-Smad3/Smad3, Smad7, and TGF-β protein levels. The band intensities (**b**,**c**,**e**–**h**). Data represent mean ± SD from triplicate wells. Statistical significance was assessed using one-way ANOVA. * *p* < 0.05, ** *p* < 0.01, *** *p* < 0.001 compared with the only UVB-exposed group; ns indicates no significant difference.

UVB irradiation affects HYAL1 and HAS2 expression, influencing skin hydration and ECM integrity [25]. As shown in Figure 7a–c, treatment with 100 μg/mL LF reduced HYAL1 expression by 67.93% and increased HAS2 by 88.32%. Additionally, UVB irradiation alters the TGF-β/Smad signaling pathway. LF treatment restored TGF-β, phosphorylated Smad2, and Smad3 expression by 49.76%, 27.76%, and 47.57%, respectively, while reducing UVB-induced Smad7 expression by 57.30%, indicating a protective effect against UV-induced damage (Figure 7d–h).

## 3. Discussion

Shikonin and lithospermic acid, the principal bioactive components of *Lithospermum erythrorhizon*, have demonstrated various skin-protective effects, including anti-inflammatory, antioxidant, and wound-healing properties [26]. Similarly, forsythiaside, the predominant active compound in *Forsythia suspensa*, exhibits antimicrobial, anti-inflammatory, and antioxidant activities, contributing to the prevention and management of skin disorders [27]. Although both natural products are well-documented individually for their dermatological benefits, studies investigating their combined effects remain limited. Thus, this study aimed to explore the protective and anti-photoaging effects of *Lithospermum erythrorhizon* and *Forsythia suspensa* on UVB-induced skin damage and aging.

Skin aging, particularly photoaging resulting from ultraviolet radiation, is a significant contributor to overall skin deterioration. UVB exposure induces excessive ROS generation, triggering oxidative stress and subsequent damage to cellular components, including DNA, lipids, and proteins [28]. This oxidative damage accelerates skin aging processes such as collagen degradation and elastin fragmentation [29]. Polyphenols and flavonoids, known for their potent antioxidative properties, can mitigate these effects by scavenging ROSs, enhancing the activity of endogenous antioxidant enzymes, and inhibiting MMP activation, thereby preserving the skin structure and delaying the photoaging process [30]. In this investigation, the Lagerstroemia floribunda extract demonstrated substantial antioxidant effects, with its total phenolic and flavonoid contents quantified as 350 ± 4.59 mg gallic acid equivalents per gram and 313 ± 7.09 mg quercetin equivalents per gram, respectively (Table 1), alongside robust radical-scavenging effects (Figure 2).

In our study, it is important to emphasize that, particularly in the ABTS assay (Figure 2b), LF exhibited lower antioxidant activity compared to the positive control AA, showing an inhibition rate of approximately 79.8%. This result stands in stark contrast to the findings from the DPPH and SOD-like activity assays, which demonstrated notably higher antioxidant potential. These observations suggest that while LF may have a limited capacity to reduce ABTS radicals, its mechanism of action may be less effective for ABTS radical scavenging. However, LF demonstrated excellent efficacy in neutralizing free radicals and superoxide anions (O_2_^−^), as supported by the results seen in Figure 3b,c. Despite this, when compared to the strong antioxidant AA, LF exhibited similar or lower antioxidant activity, highlighting the need for further studies employing additional assays and mechanisms to fully assess LF’s antioxidant potential.

Targeting the ROS-mediated ERK/AP-1 signaling pathway, a key driver of ECM degradation, further represents a promising therapeutic strategy against photoaging. Differential effects of LF and ascorbic acid on ERK/AP-1 signaling were observed, particularly with regard to c-Jun phosphorylation. While ascorbic acid robustly inhibited c-Jun phosphorylation, LF exhibited a more modest effect. This suggests that LF may modulate ERK/AP-1 signaling through alternative or indirect mechanisms, likely attributable to its complex bioactive composition. These findings warrant further investigation to elucidate the specific pathways by which LF influences c-Jun phosphorylation and overall ERK/AP-1 signaling in keratinocytes (Figure 6).

Collagen homeostasis is critical to skin structural integrity and elasticity, with MMPs playing a central role in collagen degradation. MMP-1 (interstitial collagenase) initiates the cleavage of type I and III collagen, which is further degraded by gelatinases MMP-2 and MMP-9. MMP-3 (stromelysin-1) exacerbates collagen degradation by activating latent pro-MMPs, such as pro-MMP-1 and pro-MMP-9 [31,32]. Together, these enzymes disrupt the ECM, leading to decreased skin elasticity, wrinkle formation, and sagging [33]. Our findings demonstrated that treatment with Lagerstroemia floribunda extract (100 µg/mL) significantly inhibited collagen degradation (Figure 5). Additionally, TIMP-1 and TIMP-3, play an essential role in regulating MMP activity and maintaining ECM stability. However, aging and UVB exposure disrupt the MMP/TIMP balance, favoring ECM degradation. Notably, our results showed that TIMP-1 and TIMP-3 levels increased by 64.52% and 94.28%, respectively, following treatment with the extract, as observed in Figure 4a and Figure 5f,g. This finding underscores the potential of LF extract in preserving ECM integrity through MMP/TIMP modulation.

Despite these results, when compared to the potent antioxidant AA, the efficacy of LF appears to be limited. This can be attributed to the fact that LF is a plant extract with a complex composition, and its bioactive compounds may exert effects through various mechanisms, some of which might even oppose each other. In contrast, AA is a well-characterized potent antioxidant that directly modulates these markers through well-established pathways. Moreover, LF, being a natural extract, likely contains diverse bioactive compounds that target a broad range of cellular processes, thereby producing a more generalized impact, unlike AA’s more focused action. Additionally, differences in effective concentrations, cellular uptake, and bioavailability between LF and AA may contribute to the observed discrepancies in the expression levels of TIMP-1, HYAL1, and PIP1. Future studies aimed at isolating and characterizing individual compounds within LF could provide valuable insights into their specific roles in ECM regulation and help clarify the underlying mechanisms behind these observed differences.

Chronic UVB exposure suppresses TGF-β signaling, resulting in decreased synthesis of procollagen type I peptide and the manifestation of photoaging symptoms, including wrinkles, loss of elasticity, and skin dryness [34]. Previous studies have suggested that activation of the TGF-β/Smad signaling pathway can ameliorate UV-induced skin barrier dysfunction. HaCaT cells can influence the breakdown of ECM components, including collagen, by modulating the expression of MMPs such as MMP-1, MMP-2, and MMP-9 through TGF-β/Smad signaling. These MMPs degrade collagen fibers in the ECM, contributing to skin remodeling, especially in response to environmental stressors like UV exposure. Activation of the TGF-β/Smad pathway can increase the expression of these MMPs, thereby indirectly affecting collagen homeostasis in the skin. Choi et al. reported that HaCaT cells recovered from UVB-induced damage by regulating the TGF-β/Smad pathways [11].

The present data were consistent with those from this study. In this study, LF extract modulated the TGF-β/Smad signaling pathway in UVB-irradiated HaCaT keratinocytes, significantly increasing the phosphorylation of TGF-β, Smad2, and Smad3 by 49.76%, 27.76%, and 47.57%, respectively, while downregulating Smad7 expression by 57.30% in a dose-dependent manner (Figure 7). These findings highlight the extract’s potential to mitigate UVB-induced disruptions in TGF-β/Smad signaling and promote skin barrier repair. Despite the results observed in this study, it is important to note that our research focused solely on the expression in HaCaT cells, a keratinocyte line, rather than in fibroblasts, which are primarily responsible for collagen synthesis and degradation. As such, the findings from HaCaT cells alone may not provide sufficient evidence to fully support our claims. Future research should aim to investigate the effects of LF extract on collagen synthesis and degradation in adult dermal fibroblasts to better elucidate its role in skin health.

The ECM, which includes fibrous proteins such as collagen and elastin, is crucial for skin elasticity and hydration. HA, a key component of the ECM, enhances its structural integrity by interacting with collagen and elastin. Maintaining HA levels through upregulation of HAS gene expression to stimulate HA synthesis or inhibition of HYAL activity to prevent HA degradation is vital for preserving skin elasticity [15]. Our study revealed that treatment with LF extract significantly enhanced HA synthesis by upregulating HAS2 expression (88.32%) and reduced HYAL1 activity (67.93%), effectively restoring the balance between HA synthesis and degradation in UVB-irradiated HaCaT cells (Figure 4b and Figure 7a–c). These results underscore the extract’s ability to protect against UVB-induced ECM damage and maintain skin elasticity.

**Figure 8:**
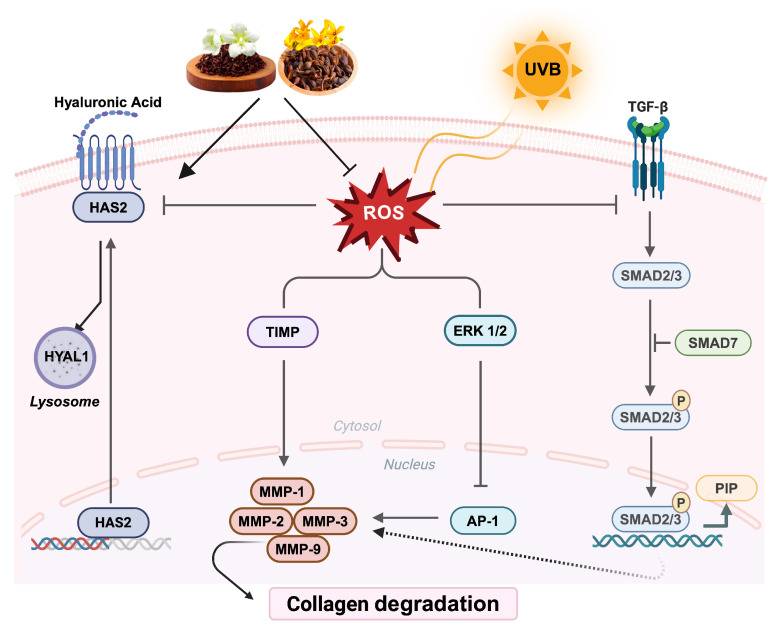
The reparative potential of LF in UVB-exposed HaCaT cells. LF demonstrated significant antioxidant properties by reducing ROS formation and inhibiting the expression of MMP-1, MMP-2, MMP-3, and MMP-9, which are enzymes linked to collagen degradation. Additionally, LF regulated the TIMP expression, contributing to the inhibition of MMP activity. LF also modulated the HAS2/HYAL1 signaling axis, essential for skin hydration, and activated the TGF-β signaling pathway, promoting collagen synthesis. These effects suggest that LF plays a key role in mitigating UVB-induced damage and enhancing the skin’s reparative mechanisms.

Based on these results, LF demonstrated protective effects against photoaging by inhibiting MMPs, which are crucial in collagen degradation. It also modulated TGF-β signaling, which is essential for collagen synthesis, by increasing phosphorylation of Smad2 and Smad3, while downregulating Smad7 expression in a dose-dependent manner. Furthermore, LF was confirmed to stimulate HA synthesis and maintain ECM balance through TIMP regulation (Figure 8). However, despite these findings, the application of LF remains unclear, particularly in the context of TGF-β signaling. The increase in TGF-β expression in HaCaT cells (keratinocytes) does not directly suggest action on collagen synthesis, as it is fibroblasts that predominantly govern collagen production in the dermis. Additionally, while LF stimulated HA synthesis, it likely involved not only HAS2 but also HAS1 and HAS3, which means further validation is needed to fully substantiate our claims.

## 4. Materials and Methods

### 4.1. Chemicals

HPLC standards (chlorogenic acid and (−)-epicatechin) and MTT were purchased from Sigma-Aldrich (St. Louis, MO, USA). DMSO and organic solvents (chloroform, isopropanol, methanol, and ethanol) were obtained from Sigma-Aldrich, Samchun Chemical (Seoul, Republic of Korea), and Daejung Chemical (Siheung, Republic of Korea). Inorganic salts were from Sigma-Aldrich, and silica gel was from Merck (Rahway, NJ, USA). DMEM and FBS were supplied by Hyclone (Logan, UT, USA), and Antibiotic-Antimycotic was from Gibco (Grand Island, NY, USA).

### 4.2. Sample Preparation

The dried powder of 300 g of Crataegus laevigata was obtained from Daelim Herbal Medicine (Jecheon, Chungcheongbuk-do, Republic of Korea) and extracted twice at room temperature for 24 h using a 1:10 (*w*/*v*) ratio of 30% ethyl alcohol. After extraction, the solution was filtered through Whatman filter paper (Little Chalfont, Buckinghamshire, USA), and the solvent was evaporated under vacuum. *Lithospermum erythrorhizon* and *Forsythia suspensa* extracts, mixed in a 1:1 ratio, were designated as LF. A voucher specimen of the plant material was deposited at Kyung Hee University Global Campus (Voucher Specimen NO: SWF2022-0322, Yongin, Gyeonggi-do, Republic of Korea).

### 4.3. HPLC Analysis

The high-performance liquid chromatography (HPLC) analysis was performed using a Dionex Chromelon TM system, which included P580 pumps and UVD100 detectors from Thermo Fisher Scientific (Waltham, MA, USA). The LF sample was prepared at 5 mg/mL in 50% methanol. Standard solutions of Lithospermic acid, Shikonin, and Forsythoside A were dissolved in 50% methanol at concentrations ranging from 3.125 to 100 µg/mL. The mobile phase consisted of a 3:1 mixture of water and acetonitrile, with 1% formic acid. The acetonitrile concentration was increased gradually from 5% to 90% over a 26 min period. Separation was performed on a Waters (Milford, MA, USA) 120 ODS-AP column (250 mm × 4.6 mm, 5 µm particle size), at a flow rate of 1 mL/min and an injection volume of 10 µL.

### 4.4. Assessment of Total Phenolic and Flavonoid Contents

TPC in LF was quantified using the Folin–Ciocalteu reagent, a colorimetric assay that measures the reducing capacity of phenolic compounds. Additionally, the TFC was determined using the aluminum chloride (AlCl_3_) method, which relies on the formation of a complex between flavonoids and AlCl_3_, resulting in a color change proportional to the flavonoid concentration.

### 4.5. Evaluation of Antioxidant Activity

The antioxidant potential of LF was evaluated through DPPH and ABTS radical scavenging assays. In the DPPH test, LF concentrations ranging from 31.25 to 1000 μg/mL were combined with DPPH solution and incubated at 37 °C for 30 min, with absorbance read at 595 nm. For the ABTS assay, LF (31.25–1000 μg/mL) was mixed with ABTS solution and incubated for 10 min at 37 °C, and absorbance was measured at 405 nm. Ascorbic acid served as a positive control, and radical scavenging activity was calculated. The formula used to determine the inhibition of DPPH and ABTS radicals is as follows:

% of radical inhibition = (OD0 − ODx)OD0 × 100

where OD0 is the absorbance of the negative control; ODx is the absorbance of various tested LF and ascorbic acid concentrations.

### 4.6. Culture and Treatment HaCaT Cells

HaCaT cells, derived from human keratinocytes, were obtained from the Korean Cell Line Bank (Seoul, Republic of Korea). The cells were cultured in DMEM (Hyclone Laboratories Inc., Logan, UT, USA) supplemented with 10% FBS and 1% penicillin-streptomycin at 37 °C in a 5% CO_2_ incubator. Upon reaching 70–80% confluency, the cells were exposed to UVB (80 mJ/cm^2^). After washing with PBS, they were treated with serum-free medium containing LF (10, 50, or 100 μg/mL) or ascorbic acid (10 μM) as a control.

When the cells reached 80% confluency, they were incubated with 10 μg/mL, 50 μg/mL, or 100 μg/mL of LF, or 10 μM AA, diluted in serum-free DMEM for 1 h. After incubation, the cells were washed twice with 1× PBS, and 5 mL of 1× PBS was then added. Subsequently, the cells were exposed to UVB radiation at a dose of 80 mJ/cm*^2^*, requiring an exposure time of 80 s, using the Bio-Link BLX-312 system (Vilber Lourmat GmbH, Marne-la-Vallée, France). Following irradiation, the cells were washed twice with 1× PBS and incubated with serum-free DMEM containing LF or AA for 24 h.

### 4.7. Cell Viability

Following treatment, cells were incubated with 1 mg/mL MTT for 12 h. The medium was removed, and formazan crystals were dissolved in DMSO. Absorbance at 595 nm was measured using a FilterMax F5 microplate reader (Sunnyvale, CA, USA).

### 4.8. ROS Inhibition Assay

HaCaT cells were exposed to UVB (80 mJ/cm^2^) and incubated with test samples for 24 h. Afterward, the cells were stained with 30 μM DCFH-DA for 30 min at 37 °C, washed with PBS, and intracellular ROS levels were measured using a fluorescence microplate reader (Molecular Devices FilterMax F5, Sunnyvale, CA, USA).

### 4.9. ELISA

The levels of TIMP-1, HAS2, HYAL 1, and PIP in the supernatant of the photoaging model were quantitatively assessed using commercially available ELISA kits. The experimental procedures followed the manufacturer’s instructions precisely.

### 4.10. RT-PCR

After UVB exposure (80 mJ/cm^2^) and 24 h sample treatment, RNA was extracted using a TRIZOL reagent (Invitrogen Life Technologies, Carlsbad, CA, USA). PCR amplification was carried out with a premix (Bioneer Co., Daejeon, Republic of Korea), and RT-PCR was performed using a Veriti Thermal Cycler (Waltham, MA, USA). The final PCR products were separated via agarose gel electrophoresis. Primer sequences for MMPs are listed in Table 2.

### 4.11. Western Blot Analysis

Cell lysates were obtained by incubating sensitized cells in RIPA buffer for 1 h, followed by centrifugation to collect the supernatant. Protein concentrations were determined using a BCA assay kit (Thermo Scientific, USA). SDS-PAGE was performed for protein separation, and proteins were transferred to PVDF membranes (Bio-Rad Laboratories, Hercules, CA, USA). After an overnight incubation with primary antibodies at 4 °C, membranes were exposed to secondary antibodies for 1 h. Chemiluminescent detection was performed with ECL reagents, and protein expression levels were analyzed using ImageMaster™ software version 2.0 (Amersham Pharmacia Biotech, Piscataway, NJ, USA). β-Actin was used as a loading control for normalization.

### 4.12. Statistical Analysis

Data are expressed as mean ± SD. Statistical differences were assessed using one-way ANOVA in GraphPad Prism 9.0. A *p* < 0.05 was considered statistically significant. Significance was denoted by * *p* < 0.05, ** *p* < 0.01, and *** *p* < 0.001 compared to untreated cells and UVB-exposed cells.

## 5. Conclusions

The present study demonstrated that LF exhibits a high content of polyphenols and phenolic compounds, which confer potent antioxidant activity. Additionally, LF was shown to inhibit ROSs in a dose-dependent manner, mitigating oxidative stress. The compound also demonstrated inhibitory effects on the MMP family, enzymes involved in the degradation of collagen and elastin. Moreover, LF enhanced the TGF-β signaling pathway, which is crucial for the synthesis of type I procollagen. Further, it modulated the expression of HAS2 and HYAL1, enzymes involved in the synthesis and degradation of HA, respectively, thus influencing skin hydration. These findings suggest that the combination of *Lithospermum erythrorhizon* and *Forsythia suspensa* may offer benefits as natural bioactive agents. In conclusion, LF demonstrates significant potential as a biological agent for protecting the skin from photoaging, indicating its potential as an active ingredient in dermatological and cosmetic formulations. Future research, including in-depth substance analysis, in vivo studies, and clinical trials, is required to further assess the therapeutic efficacy and safety profile of LF.

## Figures and Tables

**Table 1:** Quantification of the total polyphenol and flavonoid content in LF.

	**TPC (mg GAE/g Extract)**	
	Deuterium-depleted water	Dimethyl sulfoxide
LF	350.48 ± 4.59	1340 ± 2.19
	**TFC (mg QE/g Extract)**	
	Deuterium-depleted water	Dimethyl sulfoxide
LF	313.2 ± 7.09	144.2 ± 2.03

Data are expressed as the mean ± SD.

**Table 2:** Oligonucleotide primers used for RT-PCR.

NCBI Accession Code	Primera		Sequence (5-3)
NM_001145938.2	Human MMP-1	Sense	TGC GCA CAA ATC CCT TCT AC
		Antisense	TTC AAG CCC ATT TGG CAG TT
NM_004530.5	Human MMP-2	Sense	CCC GAG GTT GGA CCT ACA AG
		Antisense	CTT CCC CGT CAC CTC CAA TC
NM_002422.5	Human MMP-3	Sense	CCC GAG GTT GGA CCT ACA AG
		Antisense	CTT CCC CGT CAC CTC CAA TC
NM_004994.3	Human MMP-9	Sense	TGT ACC GCT ATG GTT ACA CT
		Antisense	GGC AGG GAC AGT TGC TTC AG
XM_011526432.2	Human GAPDH	Sense	ACC ACA GTC CAT GCC ATC AC
		Antisense	CCA CCA CCC TGT TGC TGT AG

Primer design based on the NCBI/Primer-Blast tool with standard parameters.

## Data Availability

The data presented in this study are available in this paper.
